# Implementation and evaluation in low intensity intervention programs from the CONNECT perspective of mixed methods: Application in a case of an autistic child

**DOI:** 10.3389/fpsyg.2022.998658

**Published:** 2022-10-13

**Authors:** Eulàlia Arias-Pujol, Marina Mestres, Júlia Miralbell, Natalia Bachs, M. Teresa Anguera

**Affiliations:** ^1^Faculty of Psychology, Education and Sports Sciences Blanquerna, Ramon Llull University, Barcelona, Spain; ^2^Carrilet Research and Education Center, Barcelona, Spain; ^3^Faculty of Psychology, Institute of Neurosciences, University of Barcelona, Barcelona, Spain

**Keywords:** mixed methods, case study, polar coordinate analysis, CONNECT, autism, psychoanalytic psychotherapy

## Abstract

There has been a comprehensive development over the last few years of low intensity intervention programs that are implemented within a user context and that are made up of everyday life activities, and it has been necessary to adapt the necessary methodological channels in order to guarantee an adequate resolution pathway. The mixed method perspective offers a suitable framework, and observational methodology – in itself considered mixed method – is appropriate for studying the implementation and evaluation of low intensity intervention programs, allowing the development of the QUAL-QUAN-QUAL stages that correspond to the connect integration pathway of mixed methods. In this work it was applied to a single case, in a low intensity intervention, retrieving valuable information obtained, but systematizing it and applying quantitizing to the qualitative data that was treated quantitatively in a rigorous manner. The aim was to analyze the psychotherapist-patient interaction in psychoanalytic psychotherapy, in which we sought to identify which of the therapist’s techniques stimulated actions of reciprocal social interaction in the child, and which techniques inhibited non reciprocal social interactions. The observational design was nomothetic, follow-up, and multidimensional. The patient was a 4-year-old boy with a diagnosis of severe autism spectrum disorder. We used an ad hoc observation instrument combining a field format and a category system. Interobserver agreement was analyzed quantitatively by Cohen’s kappa using the free QSEQ5 software program. Polar coordinate analysis was carried out using the free program HOISAN 2.0. Polar coordinate analysis allows us to obtain an inter-relational map of the connections detected between focal behavior established in each case and the different categories. The results provide objective evidence – backed up by the application of polar-coordinate-based data analysis – that within a framework of psychoanalytic psychotherapy, the techniques of “verbalization” and “vocalization” significantly activate reciprocal social interaction behaviors and inhibit non-social reciprocal behaviors in a child with severe autism spectrum disorder with no language. On the other hand, direct gaze promotes the child’s withdrawal. The results are of key importance as they show the therapist behaviors most useful for promoting social interaction in a child with severe autism.

## Introduction

It is an undoubtedly complex task to make decisions about the implementation and evaluation of intervention programs which, in any case, should be conditional on the applied methodology. The structural dimensions of the evaluation of a program are established in scientific literature (Chacón et al., 2000; Chacón-Moscoso et al., 2002, 2013, 2014, 2021), and the existing correspondence between these dimensions and methodological quality in the chosen procedural option is extensively relevant.

The broad definition of the aim of intervention program evaluation leads to judgments concerning the value of such programs or of some of their elements (Anguera and Chacón-Moscoso, 2008; Chacón-Moscoso et al., 2013). It has been observed that the intensity of such intervention can be extremely varied depending on the situation, taking into account that it will have considerable bearing on the procedure to be followed. It is precisely for this reason that minimum basic and common aspects have been specified that must be stated in the evaluation report of any program, regardless of its approach (Chacón-Moscoso et al., 2013), and shape a formative and summative evaluation in continual interaction, throughout its phases.

In the last few years, it has been established that the greatest shortcomings in the implementation of intervention programs are procedural, and that some of these have barely been addressed. This is mainly due to the complex nature of the reality, with many superimposed levels, and the fact that individuals or collectives who experience the actions of an intervention program may be heterogeneous. Furthermore, the dynamic of the processes is not uniform, making it difficult to collate data in a way that fulfills the requirements demanded by rigor.

In this sense, we are aware of the need to pay special attention to the implementation and evaluation of bespoke intervention programs for each specific case, aiming for an increased effectiveness in the actions carried out, without disturbing the daily life of the user, as an essential leitmotif. This special attention implies that the intervention itself blends into daily life, searching for an alternative to traditional conventionality. In this study, we focus on a low *level of intervention intensity*, an expression coined by Anguera (2008, p. 154), in the face of an “incessant increment in cases in which intervention programs are implemented without the imposition of instructions, and in contexts that are natural and/or habitual for the users of the program, taking advantage of activities that are spontaneous and/or everyday for them.” There have been numerous studies over the last few decades that have dealt with the handling of an intervention in the habitual context of a user, with the different programs having very diverse aims (Weil, 1985; Bryant and Bickman, 1996; Lapresa et al., 2020), and with beneficial effects in terms of the ‘normalization’ of the user’s life (Dvoskin and Steadman, 1994; Roustan et al., 2013; Alcover et al., 2019), studying the interaction in the therapeutic conversation (Arias-Pujol and Anguera, 2017, 2020a,2020b; Del Giacco et al., 2020).

In this study we present a low intensity level program – a psychoanalytic psychotherapy intervention for a child with severe autism. It is carried out in a natural context and stems from the child’s predominantly sensory spontaneous behavior, disconnected from social interaction. The therapeutic technique seeks to help children establish connections between their sensory experiences, their emotions, language and thought, following the approach developed by the prestigious Coromines (Coromines, 1991; Viloca, 2011; Farrés et al., 2020). It is a dyadic program based on the verbal and non-verbal communication between the child and the therapist. An adequate evaluation requires the flexibility and scientific rigor of systematic observation from the mixed method perspective.

In Chacón-Moscoso et al. (2021) an adaptation was carried out of the structural dimensions of low intensity level designs when observational methodology is applied (Anguera, 2003). This is characterized as a scientific method that allows for the study of spontaneous behavior in habitual contexts. Its defining elements are the following: (1) delimiting the aim from a prior needs analysis; (2) building the program depending on the theoretical framework and the intervention context; (3) guaranteeing its usability and sufficiency; (4) proposing a suitable design; (5) implementing the actions of the program in such a way as to obtain a diachronic record throughout the recommended monitoring; (6) building a non-standard instrument adapted to the object of study; (7) systemizing the information; (8) controlling data quality; and (9) assessing the program with adequate analysis techniques. It should be highlighted that in the last two decades the mixed method perspective has been developed exponentially, generating an important cross-fertilization process in terms of procedure.

We would like to point out one final aspect to be developed, relating to the fact that we are dealing with a case study. Here we attempt to transform something that has acquired negative connotations in the literature of the last few years into a worthwhile opportunity that makes a rigorous, intensive study of human behavior possible.

### Contributions of the mixed method perspective in the evaluation of low intensity programs

The mixed method perspective has had a significant impact all over the world throughout the last few years, awakening an exponential interest and generating an extremely high volume of scientific production. As is widely known, a harsh dialectic was produced over decades between those in favor of qualitative and quantitative approaches. Almost a quarter of a century ago, Newman and Benz (1998), set out to explore the interactive qualitative-quantitative *continuum* in research. They considered false and without foundation the dichotomy that many other authors have tried to show, by presenting both options as opposing paradigms and refusing to accept that both qualitative and quantitative strategies are always found in any study. Their approach, focusing on the feedback between qualitative and quantitative analysis, can be considered as an accurate forerunner to the current, complex state of the issue (Anguera, 2022), that forces us to be vigilant whilst moving forward.

Observational studies were initially qualitative, proof of which lies in the main works published in the 1970s and 1980s (Weick, 1968; Hutt and Hutt, 1974; Mucchielli, 1974; Anguera, 1979; Martin and Bateson, 1991), whose aim was to capture the reality in a descriptive way just as it was produced – but which our approach challenges – which led to the belief that qualitative methodology fit with the first half of the process, whilst quantitative methodology should be used in the second half of the process (Sánchez-Algarra and Anguera, 2013). Furthermore, in the last few years things have gone a step further, with the consideration that observational methodology was in itself mixed method (Anguera and Hernández-Mendo, 2016; Anguera et al., 2017) – also in indirect observation (Anguera et al., 2018b) –, and proposing a form of quantitizing as an integration path (Anguera et al., 2020).

The specification of *quantitizing* in observation methodology is founded on Creswell and Plano Clark’s, 2011 contribution (3rd ed., 2011), that we especially value:

There are three ways in which mixing occurs: *merging* or converging the two datasets by actually bringing them together, *connecting* the two datasets by having one build on the other, or *embedding* one dataset within the other so that one type of data provides a supportive role for the other dataset. (p. 7) (the underlining is ours).

This *mixing*, in the CONNECT option, taken both literally and from a wider perspective (Anguera, 2022), is a strong basis for carrying out a reconsideration of *quantitizing* that fits very well within observational methodology.

Indeed, in literal terms, “*connecting* the two datasets by having one build on the other,” would imply that one dataset can give rise to another via its transformation. Such a transformation must guarantee the maintenance of its informative quality, whilst modifying the appearance. From a wider perspective, *connecting* allows the alternation of the QUAL-QUAN-QUAL stages; this legitimizes the generic mixed method approach, such that a total integration of qualitative and quantitative elements is achieved (Anguera et al., 2020).

The materialization of *quantitizing* takes place between the first QUAL stage and the QUAN stage (Anguera et al., 2020; Izquierdo and Anguera, 2021), and is accomplished from two fundamental decisions (Anguera, 2017, 2022): (1) establishment of the design dimensions (or response levels, or criteria) (Weick, 1968), that can be deployed in sub-dimensions on different levels; and (2) proposing segmentation criteria of the observed behavior episodes (or textual material, in indirect observation) in observation units (Anguera, 2020, 2021). The two decisions (dimensions and observation units) were developed later, both in direct observation (Anguera, 2017) and indirect observation (Anguera, 2021).

Once these decisions have been taken and the *ad hoc* observation instrument built, the record is created, made up of qualitative data, and will preferably be structured in the form of a code matrix, with columns containing the dimensions (or, where appropriate, the sub-dimensions of the most molecularized level); with each row of the matrix containing the codes corresponding to the co-occurrence of the different dimensions in each unit of behavior. This matrix is essential for the process of *quantitizing* the qualitative data.

Once the data matrix has been obtained, its quality must be controlled via one of the concordance/agreement indexes (Blanco-Villaseñor and Anguera, 2000); and once this is done then the quantitative analysis of the systematized qualitative data is possible (and, among others, the analysis of polar coordinates), thus allowing a complete integration between qualitative and quantitative elements.

Taking this reconsideration into account, the innovative form of *quantitizing* in the implementation and evaluation of low intensity programs implies important methodological benefits (Anguera, 2022). The mixed method greatly vitalizes the collation, management and analysis of information obtained via observation, which previously played a trivial, superficial and incoherent role. An important strengthening of range is achieved from observational methodology (both direct, as in this study, and also indirect), due to it being considered in itself mixed method; hence, in the Introduction we refer to the process of cross-fertilization.

We strongly defend this approach (Anguera, 2022), that has been progressively structured over the last two decades, without evading the attraction of the mixed methods being developed (Sánchez-Algarra and Anguera, 2013; Portell et al., 2015; Chacón-Moscoso et al., 2019; Anguera et al., 2020; Izquierdo and Anguera, 2021); these differing from multi-method studies (Anguera et al., 2018a), constituting an already consolidated culture of research (Anguera et al., in press).

### The case study in the evaluation of low intensity programs

The case study has traditionally been considered marginal and with little convening power; in addition to renowned authors such as Gerring (2004, p. 341) stating that “the case study survives in a curious methodological limbo.”

It is generically accepted as a study that is intensive, detailed, in-depth, centered on one “case,” and focusing on “the particular”; and undoubtedly influenced by the context in which it is located and the theoretical framework that covers it (Anguera, 2018). The case study implies an “intensive study of a single unit for the purpose of understanding a larger class of (similar) units … observed at a single point in time or over some delimited period of time” (Gerring, 2004, p. 342), provided that it is carried out with guarantees of suitability (Kuyken et al., 2005; Flinn et al., 2015).

For Stake (1994), the case study does not represent a methodological option, but an object of study option. However, Campbell, in his *Foreword* to Yin (2014), and the thematic development itself of this author, do openly consider it a research method. There have been many dissonant voices in different publications (Anguera, 2018), and there is no consensus concerning its methodological range, although there is a majority who do not consider it a methodology. Tight (2010, p. 329) asks the following question: “Case study is widely referred to and applied within social research, but its status remains unclear. Is it a method, a methodology, a strategy, a design, an approach, or what?”.

In our view (Anguera, 2018), it is not a methodology, but it is possible to apply diverse methodologies to a single case. Aside from existing typologies (Stake, 1994; Thomas, 2011; Yin, 2014), the logic of the single case is intra-case by nature (Hilliard, 1993) and permits the consideration of a diachronic perspective, whilst at the same time emphasizing the richness of the context in the real world in which the phenomenon is produced.

There is obviously an inherent weakness in the case study, relating to the non-replication of results, which is totally logical according to its own aim. Its focus of attention is found precisely in the opposing situation, focused on the results of one single case which is studied in depth, and is shown in this study. In our view, the strength of a methodology that is appropriate and adapted to the characteristics of the case study and the profile of the case itself compensates for this weakness.

The methodological criticism that the case study has received focuses essentially on the problem of a lack of representativeness; although the case is an individuality with an initial presumption of singularity, which is studied intensively. As Sandelowski states (Sandelowski, 1996, p. 527), “the analyst works to discern what elements comprise the case and, more importantly, the way they come together uniquely to characterize the case,” which suggests that researchers should establish the profile of the case (Anguera, 2018).

The profile of the case we present below corresponds to that of a child with severe autism. The therapist provokes interaction by initiating communicative turns of protoconversation, verbalizing sensations and emotions that the child feels or could feel, and also dramatizing them (Trevarthen and Aitken, 2001; Viloca, 2003; Alcàcer and Viloca, 2014). Unlike other studies in which we narrate the intervention and clinical improvement of the patient (Arias-Pujol et al., 2015a,b), on this occasion we are interested in focusing on the technique used by the therapist. The suitable methodology is systematic observation, this being equipped with design that supports an intensive approach in the study of perceptible behaviors; carrying out continuous recordings throughout the observation sessions, and being able to manage behaviors arising from different dimensions/sub-dimensions, some specific to the therapist’s role and others to the child’s actions. In other words, we transform into a methodological opportunity that which has traditionally been the biggest weakness in case studies.

For their part, Edwards et al. (2004) state that they do not wish to differentiate between qualitative and quantitative approaches in the case study. As Stake (1994, 1995) indicates, the case study can be qualitative (Hilliard, 1993), or quantitative (Kent, 2009), or a combination of both (mixed methods are currently conceived within a continuum between the qualitative and quantitative poles); although he leans more toward a clear qualitative predominance (Stake, 2005). Our proposal, in line with the above, involves taking advantage of the appearance of a third way, that of the aforementioned mixed methods, which places us in a privileged position of integrating qualitative and quantitative elements.

### Aim

To analyze, from the mixed methods perspective, a case study consisting of low intensity psychotherapeutic intervention focused on a child with a diagnosis of severe autism who interacts with the psychotherapist; and in which we aim to identify which actions on the part of the therapist stimulate social interaction from the child.

### Design

Observational methodology was applied. The observational design is Nomothetic/Follow-up/Multidimensional (N/F/M) (Anguera et al., 2001; Sánchez-Algarra and Anguera, 2013): nomothetic because we studied the interaction between therapist and autistic child, with inter-session follow-up (three sessions) and intra-session follow-up (because each session was recorded continuously from start to finish); and multidimensional since the complexity of the aim required the application of various dimensions that were included in the observation instrument.

### Participants

There were two participants:

The patient was a 4-year-old child with a diagnosis of autism spectrum disorder (ASD), according to the clinical criteria of DSM-5 (American Psychiatric Association, 2015), of a severe type according to the results obtained from the ADOS (Autism Diagnostic Observation Schedule; Lord et al., 2000). The child had no language, although did emit sounds and some syllables forming echolalia.

The therapist was a clinical psychologist with training and experience in psychoanalytic psychotherapy with children.

In accordance with the principles of the Declaration of Helsinki and the Ethical Code of the General Council of the Official College of Psychologists of Spain, the child and the child’s family were informed that they were being filmed. They were shown the location of the video cameras, which were positioned discretely to minimize reactivity bias. Written informed consent was also obtained from the parents of the minor.

### Program intervention plan

The psychotherapeutic intervention was designed for a child with severe ASD. It consisted of 20 weekly sessions of 45 min in length, and was focused on stimulating reciprocal social interaction in the child, arising from the relationship. We adapted the psychoanalytic technique described by Coromines that promotes a process of differentiation and of interest in the “Other” via a shared emotional experience between therapist and patient (Viloca, 2003; Farrés et al., 2020). The sessions took place in Carrilet Treatment Center (Barcelona, Spain), attached to the educational and therapeutic center.

### Instruments

#### Recording instrument

All the sessions were filmed using a video camera installed in the therapy office of the Carrilet Treatment Center attached to the Educational and Therapeutic Center where the child was schooled.

#### Observation instrument

We used an *ad hoc* observation instrument, as a field format modality combined with category systems, adapted by Bachs and Arias-Pujol (Bachs, 2019) from a previous study (Arias-Pujol et al., 2015b) and recoded for this new study. The instrument has two dimensions for the child: reciprocal social interaction (RSI) and non-reciprocal social interaction (N_RSI) and 12 for the therapist (see Table 1).

**TABLE 1:** Observation instrument of ASD child and psychotherapist in psychoanalytic psychotherapy.

Name and definition of categories	Code
***Observation instrument of ASD child***	
**Non-reciprocal social interaction (N_RSI):** Actions carried out by the child in relation to an object or toward himself/herself.	
**(1) Stereotypes**: repetitive behaviors that always follow the same pattern. They can be of different types: (1.1) **Motor:** highly repetitive movements with consistent action patterns (by emotion or sensation) *Example*: jumps [on themselves], arm flapping and gesticulation, rocking. (1.2) **Vocal:** highly repetitive vocalizations with consistent intonation patterns such as humming, various vocal repetitions (By emotion or sensation) *Example*: tititi, hmmm, ah ahah, d-gæ-d-gæ-d-gæ, iiiiii, tacataca. (1.3) **Visual:** strange eye movements, such as looking sidelong at the camera, suddenly diverting the gaze after maintaining eye contact with the therapist, objects, etc. *Example*: looking sidelong from one side to the other.	MS VS VIS
**(2) Erratic Behavior**: constant wandering with no set purpose. It occurs when the child moves, walks around the office without a clear intent. When the child grasps an object when there is no subsequent functional intent. He/she takes it and then leaves it. *Example*: wandering without exploring, jumping around the room, grasping and abruptly throwing an object.	EB
**(3) Auto Sensory**: an action carried out by the child that provokes a sensation, with no exploratory functional purpose. *Example*: hitting with an object, abruptly pulling an object, continuously and persistently touching different objects, touching the face, taking a handkerchief and wiping the face with it, tracing their sneakers with the hands or the edge of the chair with the fingers, keeping one hand on the wall when walking or leaning on it with the whole body. Despite appearing as repetitive behavior, it is not considered stereotypical because: (a) the behavioral pattern is not always identical. (b) it is exploratory via sensation, and c. it is of low frequency *Covering the ear would be included due to being exploratory via sensation.*	AS
**(4) Functional Intent**: normal actions with objects, the objects are used with a coherent purpose for which they were created. *Example*: order, collect, take out, put on, take off, shake a box of blocks, take crayons from a box, open a box, arrange a chair… *In the case of continuous repetition of behaviors FI, consider the category “Solitary play (SP)”.*	INT
**(5) Gaze:** (5.1) Attentive object: when the child gazes attentively at an object. The object has entered the field of vision and is noticed. It is an action that has exploratory aspects. *Example*: “He/she takes the box with both hands + **stares at it** + turns it upside down to shake out the blocks + puts one hand inside to remove them (he/she achieves this) + continues shaking with force”. *Example*: “He/she sits down + touches the dice with his/her fingers and makes them move + “oh” + continues touching the dice with the fingers + **looks at them very attentively.**” (5.2) Blank stare: when the child remains staring at the floor, the ceiling or the wall when there is no specific object at which to direct the gaze.	AGO BS
**(6) Solitary Play**: there is an appropriate use of the toy. The relationship with the object (such as blocks for building a tower) is more structured and sequenced than in the functional intent category. This category includes those sequences that are repeated in various turns, becoming a ritualized game despite the therapist’s intervention. It also includes exploratory play, i.e., when the child manipulates and relates to the object, also permitting the predominance of sensory self-stimulation throughout the action. The child shows an interest, his/her behavior has a purpose in itself and at the same time is very sensory (wants to build the tower, wants to make it taller, wants to hit the balloon, scribble with the crayons…). *Example*: the child begins a tower + puts down two blocks + takes out a block + changes one block for another + mmmmm (SP + VS). *Example*: the child moves the trucks from one side to the other + “brumbrum” + lifts them up and leaves them on the floor, organizing them.	SP
**(7) Normal Actions**: actions that the child carries out, that are related to being in one place (with no auto sensory purpose). *Example*: gets up, sits down, turns, crouches, lifts the head, and repositions the body.	NA
**Reciprocal social interaction (RSI):** They are actions that the child carries out, bearing in mind the therapist or in response to an action by the therapist.	
**(8) Demand**: when the child addresses the therapist for the purpose of asking for something. *We differentiate between a continuum of more fusion to more differentiation ME – NOT ME* It can be: (8.1) **Instrumentalization/Instrumentalized Demand**: undifferentiated demand (no me–you difference). It occurs when the child uses the other as an object in order to achieve a purpose, in something which he/she knows how to do motively. Also when the child uses the other as an object to achieve something he/she can’t do for himself/herself. *Example*: the child wants to open the bubble container. He/she takes the therapist’s hand with both hands and moves them toward the container for the therapist to open it and then steps back waiting for T to do it (the child knows how to open it, but takes the therapist’s hand as his/her own limb). *If the instrumentalization is followed by a VS or MS, consider categories VOCD or NVD.* (8.2) **Non-verbal Demand**: when the child communicates an intention or desire and uses his/her body to achieve it; for example touching the object, showing it, pointing to it, etc. *Example*: the child looks at T and takes the bubble container from the table. He/she moves it (making a noise) and gives it to the therapist. *Example*: the child stands behind the therapist’s chair placing both hands on her/him. He/she uses force to move the therapist because he/she wants T to move. Then he/she steps back, waiting for a response. (8.3) **Vocal Demand:** a demand from the child accompanied by a vocalization, albeit stereotyped. *Example*: “He/she moves away to go and look for the balloon + takes it + gives it to T (who keeps hold of it) + guttural sound” (VS) (8.4) **Verbal Demand:** a demand from the child accompanied by verbalization or word approximation.	I NVD VOCD VD
**(9) Response to a demand** (9.1) **Active response/following proposal**: when the child responds actively to a demand or to the therapist’s stimulation proposal. *Example*: the therapist inflates a balloon, moves it (making a noise) and the child approaches and takes it very carefully. *Example*: the therapist says: “oh, have we changed?” (referring to how P has changed the blocks around) and the child looks at T and changes them back again. *Example*: the therapist: partly opens the box + “Now you can open it” + looks at P, and the child: looks at the box + bites lip + smiles + opens the box with both hands. (9.2) **No active response/no following proposal**: when the child does not respond to the demand or the therapist’s stimulation proposal. *Example*: T tries to give the child the bubbles, but P rejects them abruptly. *Example*: T: “Tc-tc-tc” (paused) while T tickles the child (from lower back up to the neck); P: Turns body forwards to avoid it + “iiii” (with a high-pitched intonation).	AF NAF
**(10) Proxemic Behavior**: the child’s trajectory with the therapist, in response or not to an action or verbalization from the therapist. (10.1) Moves closer. (10.2) Moves away.	APRO DPB
**(11) Physical Contact.** (11.1) Brief *Example*: child touches the therapist. (11.2) Maintained *Example*: child hugs the therapist, sits on T’s lap. **(12) Joint Attention**: when the child and the therapist share the same focus of attention, whether it be an object or an activity sequence. There is a shared pleasure and the action takes turns: (12.1) **Child shows** or points with the purpose of sharing the attention to an object. Thus, a shared experience with the therapist is created. Example: placing an object where the therapist can see it, holding the object in front of the therapist, pointing to an object with the aim of the therapist seeing it, or giving an object. 12.2 **Child draws.** (12.3) **Protoconversation:** a type of dialogue via vocalizations and/or verbalizations that follow turns of intervention and that make sense within the context in which they are expressed. They are different from the verbal stereotype in that there is a communicative intention. Types: (12.3.1) Vocalization/word approximation: The production has at least a vocal similarity with a word which makes sense in the context. It includes onomatopoeia. Example: Ahh! Example: “aul” (azul - *blue*). (12.3.2) Word: The production is clear. (12.3.3) Sentence approximation: 2 or more consecutive, related words. “Esto e aul” (“*This is blue*”).	BPC MPC SH DRA WA WO SAP
**(13) Gaze**: when the child’s gaze is in relation to the therapist. Eye contact. The child looks at the therapist to establish eye contact. It is categorized as EC although brief – a “fleeting glance” (Viloca, 2003).	EC
**(14) Imitation:** This category should be produced in response to a stimulation, imitation or proposal by the therapist. (14.1) Vocal – Verbal (14.2) Non-verbal	VI NVI
**(15) Facial Expression (FEx).** (15.1) Rejection: when the child shows a facial expression of displeasure, anger, upset or sighs. (15.2) Joy: when the child shows a facial expression of joy, laughs or smiles.	FEXR FEXJ
***Observation instrument of the psychoanalytical psychotherapist***	
(1) **Verbalize:** Put into words. Therapist uses language to bring the child closer to the symbolization process (following the psycho-pedagogical scheme of Dra. Coromines, 1991; Viloca, 1998, 2003). (1.1) **Describes** a behavior or an object. Suggests to the child a feeling or a desire. It puts words to the child’s behaviors, feelings, emotions, desires, and thoughts. Given the hypersensitivity of these children, it includes naming interferences and giving information from the context such as ambient noises, if the material makes noise or if an object fails. Examples: (a) Name object and sensation: “Oh! It’s a sponge, it’s hard and scratchy,” (b) Behavior: “Ah, you want it all for yourself!”, “You have seen the bubbles and left the dice,” “You have thrown them all,” (c) Desire: “Shall we blow?”, “More?”, (d) Context: “What a loud noise, huh? Tocotoco. They made noise,” “What noise do you make, huh? You hear noise outside and you make a noise, tacataca!”. (1.2) **Offers help:** the therapist verbalizes an offer of help to the child when T sees that the child needs it. Example: “Let’s see, can I help you … open the box?” + opens the box, “Do you want to open **the box?”** + pauses + “the crayons?” (1.3) **Anticipates:** the therapist anticipates actions that will be carried out soon, in the immediate or distant future *Example*: “Do you want to open the box?” + pauses + “**The crayons?**”; The therapist says: “One, two and…” singing + inhaling air + blowing the bubbles; The child takes the cup and puts it in his/her mouth. Meanwhile, the therapist says: “Let’s put some water in it” + takes another cup from the box. (1.4) **Reminds:** the therapist verbalizes actions that have been carried out previously. *Example*: T looks at the child + **“The other day Biel was singing.”** + starts to sing a melody. (1.5) **Gives support:** the therapist supports, encourages, congratulates, grants. T expresses approval of the child’s behavior with words or gestures. Example: “Well done!”, “Thank you!”, “Very good!” (1.6) **Repeats to show understanding:** the therapist repeats what the child has just said in a similar manner in order to start a dialogue, although the child doesn’t follow T’s lead, T tries to make sense of the child’s verbal communications, even though they might not be very clear. There is recognition of the child’s verbalizations. Similar to the protoconversation that takes place between a parent and baby. *Example*: the child looks at the therapist + holds up the object + “the blocks”; the therapist looks at the child + nods head + “The blocks”. (1.7) **Interpretation** (INT): the therapist verbalizes the emotion and goes further, giving meaning, a possible explanation of what the child does, says or feels. T connects what the child does with what she/he imagines the child could be feeling. T goes a step further in understanding the facts and emotional experience the child is living. Examples: The child takes a cup from the box and starts to drink. When he/she stops he/she says “hm” with a disgusted face. The therapist then laughs and says: “Ecs, is it not good?”; The child is out of camera shot, but the therapist says: “Ui, ui what a face!”; The child turns his/her head and laughs; the therapist: “Ah, you’re smiling” and also laughs.	VDE VOH VAN VREM VSUP RMU INT
**(2) Vocalization**: (2.1) Exclamatory elocution: Sounds or onomatopoeia that the therapist makes, expressing or highlighting an emotion. Examples: “Oh!”, “<Ala!”, “Oi!”, “Ehi!!”. (2.2) Sings	EE, SI
**(3) Imitation:** the therapist copies the child’s behavior. This category includes the three levels of imitation described in (annex 1): sensorial resonance, significant imitation and double material. It also includes actions in which the therapist follows the child’s activity or adds to what the child is doing (looking for a turn-taking game). Example: The child is piling up blocks and the therapist starts to pile up blocks next to him, as in taking turns (the child does not modify his/her behavior, it is the therapist who adapts). (3.1) Verbal imitation. (3.2) Non-verbal imitation.	VIT NVIT
**(4) Stimulation:** it can be verbal or non-verbal, here we do not differentiate. At times the therapist makes an active change in the setting. (4.1) **Gives:** the therapist gives the child and object to facilitate play, attract the child’s attention or initiate a game. *Examples*: The therapist says: “Shall I open the crayons?” + opens them + “There you go” + **stretches out his/her arm to give them to child;** “Here you are” + **T gives the balloons to child.** (4.2) **Shows:** the therapist shows the child an object or an action, with the aim of directing his/her attention. *Example:* “Sheets of paper” + **points to them** + sits in the chair. (4.3**) Directs attention**: the therapist directs the child’s attention toward an object, a noise or an action in order to initiate a new activity, a game, or to interrupt something repetitive. *Example*: when the therapist shakes an object (balloons, blocks, etc.) to get the child’s attention. (4.4) **Directs behavior:** when the therapist gives an order, asks for, guides, shows or encourages the child to do something in a particular way. (4.5) **Proposes:** the therapist starts an activity, expecting the child to follow or, faced with the child’s indecision, proposes a joint game with material that the child has not yet used. Or also when the child is doing nothing or performing a repetitive action. *Example*: The child moves the blocks + places a block (yellow) on the floor + takes another block (red) with one hand and with the other takes the therapist’s hand and moves it closer + “eh.” Thus, T responds: “Aaah” + takes the red block and puts it on top of the red block + “Here?”	SG SSH SDA SDB SPRO
**(5) Non-verbal Behavior (NVB).** (5.1) Facial expression: the therapist shows with a facial expression (surprise, disgust, smiles.) or the movement of hands and torso (gesture). (5.2) Gaze: We consider that the therapist tends to look at the child, but if we see in the transcript that it is specified, then we will code it.	FEXT GA
**(6) Auxiliary Functions**: facilitating actions carried out by the therapist, such as moving an object closer that the child needs, opening a bag, uncovering the box. When there is no clear demand from the child, since in that case it would be “responds to demand.” When the therapist senses the need, functioning as an auxiliary Me.	AUXF
**(7) Responds to the instrumentalized demand/proposal:** takes notice by responding to an action of demand more or less instrumentalized or to a proposal from the child.	RID
**(8) Does not respond to the instrumentalized demand:** the therapist does not agree to do what the child is expressing with a clear petition or proposal (albeit non-verbal).	NRID
**(9) Proxemic Behavior:** moving closer to or away from the child. (9.1) Closeness *Example*: T lies on the floor next to the child. (9.2) Distance *Example*: T moves away from the child.	APROT DT
**(10) Physical Contact**: the therapist touches, holds, tickles, caresses, hugs. It can be a brief moment or a maintained contact that can form part of a game or cuddles. (10.1) Brief *Example*: the therapist tickles the child, touching him/her briefly. (10.2) Maintained *Example*: T strokes hand over the child’s back, head. T holds the child in her/his lap.	BPCT MPCT
**(11) Normal Actions**: actions carried out by the therapist that are related with preserving the “*setting*,” such as adjusting her/his posture to that of the child, curling up, getting comfortable, etc.	NAT
**(12) Phatic Function**: the therapist encourages the child to continue speaking or waits for a reply from him/her, giving over her/his turn. *Example*: nods head waiting for a response + “hmhm”; Child takes the bubbles from the box; T smiles widely and assents.	PF

Adapted from Bachs (2019).

With the aim of studying the therapist-patient relationship, the sessions were broken down into units, with the adoption of a primary dialogic criterion, and a secondary criterion that differentiated verbal, vocalized and non-verbal behavior in the transcript (Anguera, 2020). Table 2 shows some fragments of the coded clinical material from sessions 9, 16, and 20.

**TABLE 2:** Fragments of the coded clinical material.

Session 9
**They are playing with a balloon which has deflated and fallen onto the floor. The child wants the T to inflate it again.** **C:** He/she bends down (NA) + picks up the balloon (INT) + turns to the T (NA) + and goes towards her/him (APRO) + makes the gesture of giving the balloon (NVD) **T:** He/she looks at the child (GA) + makes a surprised face (FEXT) + “the pink balloon?” (VDE) + picks up the balloon (RID) **C:** “Tii” (WA) + brushes the wall with his/her back (SA) + “Buh-ah!” (WA) + looks at the T (EC) + takes T’s hand that is holding the balloon and moves it towards the therapist’s mouth (I) **T:** “Oh” (EE) + “I’m going to blow up the balloon” (VAN) + inflates it (RID)

**Session 16**

**The child places blocks on top of each other, building a tower. The T attempts to join in the game.** **T:** “Oh” (EE) + “a very high tower” (VDE) + takes one of the blocks and moves it towards the child to give it to him/her (SG) **C:** “Oh” (WA) + ignores the block (NAF) **T:** “Here you are” (SG) **C:** He/she moves his/her arm and takes the T’s block with one hand (AF) and picks up another with the other hand (SP) **T:** “Very good” (VSUP) + Smiles (FEXT) **C:** “TcTcTc” (VS) + Keeps both blocks, one in each hand, and adds one of the blocks to the tower (SP) **T:** Look (GA) **C:** He/she adds the other (SP) + “TcTcTc” (VS)

**Session 20**

**Through imitation, a sort of dance is created between the child and the T of moving closer together and further apart. They are holding hands. The T imitates the child’s vocal stereotypies.** **T:** “Taaa taaaan.” (VIT) **C:** Looks at the T (EC) + Drops his/her hands and moves away toward the desk (DPB) + “Taaa taaa” (VS) **T:** He/she walks toward the other side, moving away (DT) + looks at the child (GA) + “Taaa taaa.” (VIT) + smiles (FEXT) **C:** Walks toward the T (APRO) + takes her/his hand (BPC) + twists around (SA) + takes T’s hand with his/her hand (BPC)

T, therapist; C, child.

### Procedure

#### Inter observer agreement

The inter observer agreement was calculated via Cohen’s kappa coefficient (κ) (Cohen, 1960). Data quality control was performed using the free program GSEQ. An agreement of 85.6% was obtained in the codification of the child’s behavior and of 90.4% in that of the therapist, values considered “almost perfect” according to the criteria of Landis and Koch (1977) and Bachs (2019).

### Data analysis

#### Analysis of polar coordinate

The aim is to apply the analytical technique of polar coordinate analysis, seeking a possible relationship of activation/inhibition between the behaviors of the therapist and the child that will be quantitatively calculated from the qualitative recordings carried out initially at three different points of the intervention, plotting them via vectors.

Polar coordinate analysis allows us to obtain an inter-relational map of the connections detected between the different categories. It is a robust analytical technique developed by Sackett (1980) that is based on the sequentiality of the qualitative records obtained. The focal behavior – located in the center of each of the ‘maps’ that will be created – must be identified in each analysis, and is proposed depending on the desired aim and the conditioned behaviors; these being all those about which we want to know whether there is an associative relationship with the focal behavior. The associative relationships between the focal behavior and each one of the conditioned behaviors incorporate two perspectives: prospective (from the focal behavior forwards) and retrospective (from the focal behavior backwards). We should clarify that we are not referring to a classical retrospective perspective, but to the genuine retrospectivity proposed by Anguera (1997), which has since been consolidated. The calculation required to apply the prospective and retrospective perspectives generates a huge volume of partial results, and Sackett (1980) knew how to exploit the possibilities of the *Z*_sum_ parameter proposed by Cochran (1954) as an important data reducer. The calculation of the prospective and retrospective *Z*_sum_ values, in accordance with Sackett’s approach (Sackett, 1980), allows us to obtain the values that correspond to the length of the vector and its angle, which can be graphically represented. The vector angle (that will correspond to one of the quadrants I, II, III, and IV), allows us to interpret the nature of the relationship that exists between the focal behavior and the respective conditioned behavior, while from the length of the vector we can interpret the intensity of said relationship depending on statistic significance.

We have presented all these calculations systematized in our case study (see Table 3), in such a way that the conditioned behaviors appear for each focal behavior; and for each conditioned behavior information is presented, correspondent to the quadrant in which the vector is found: prospective and retrospective values of the *Z*_sum_, ratio, length of vector (that is crucial for knowing its significance, since if it is > 1.96 it is significant, and if it is > 2.58 it is very significant), significance, and vector angle.

**TABLE 3:** Table of parameters corresponding to the analysis of polar coordinates, with the focal behavior “Verbalizes,” “Vocalizes,” “Verbal imitation,” “Nonverbal imitation,” “Stimulates,” “FEXT,” and “GA” as the center of the analysis and all the others as conditional of sessions 9, 16, and 20.

Focal behavior (therapist)	Conditioned behavior (child)	Quadrant	Prospective zsum	Retrospective zsum	Ratio	Length	Significance	Angle
**Session 9**								
Verbalizes	Erratic behavior_EB	n	−0,4	2,15	0,98	2,19	*	100,59
	Functional intent FI	i	0,22	3,07	1	3,08	**	85,9
	Attentive object_AGO	I	4,09	0,54	0,13	4,13	**	7,52
	Normal actions_NA	I	3,4	0,59	0,17	3,45	**	9,78
	Demanda_DNV	II	−1,08	4,57	0,97	4,69	**	103,27
	No active response/no following proposal_NAF	I	0,01	2,29	1	2,29	*	89,68
	Joint attention_WO	IV	1,77	−0,88	−0,44	1,98	*	333,6
Vocalizes	Stereotypes_VS	HI	−0,97	−2,46	−0,93	2,65	**	248,37
	Erratic behavior_EB	IV	3,49	−0,25	−0,07	3,5	**	355,87
	Attentive object_AGO	N	−0,81	2,32	0,94	2,46	*	109,33
	Solitary play_SP	HI	−1,83	−1,21	−0,55	2,19	*	213,42
	Instrumentalization/instrumentalized	I	1,55	2,46	0,85	2,91	**	57,68
	Demand_I							
	Demanda_DNV	I	2,6	1,8	0,57	3,16	**	34,73
	Active response/following proposal_AF	I	1,01	3,38	0,96	3,53	**	73,37
	Proxemic behavior DPB	IV	2,94	−0,43	−0,15	2,97	**	351,61
Verbal imitation	Stereotypes_VS	I	1,27	2,41	0,88	2,73	**	62,15
	Autosensorialidad AS	N	−0,79	1,89	0,92	2,05	*	112,7
	Solitary play_SP	HI	−1,74	−1,65	−0,69	2,4	*	223,53
	Demanda_DNV	i	2,01	0,5	0,24	2,07	*	13,86
	Joint attention_WA	IV	2.48	−0,85	−0,32	2,62	**	341,06
	Facial expression_FEXR	n	−0,45	2	0,98	2,05	*	102,78
Nonverbal imitation	Stereotypes VS	i	3,44	0,38	0,11	3.46	**	6,29
	Erratic behavior_EB	HI	−1,72	−1	−0,5	1,99	*	210,21
	Autosensorialidad_AS	i	1,16	2.94	0,93	3,16	**	68,39
	Attentive object_AGO	II	−0,13	1,97	1	1,97	*	93,71
	Solitary play_SP	HI	−1,55	−1,54	−0,7	2,18	*	224,78
	No active response/no following proposal_NAF	i	0,78	2,27	0,95	2,4	*	70,95
	Joint attention_DRA	i	5,66	6,96	0,78	8,96	**	50,89
	Joint attention_WO	II	−0,4	2,16	0,98	2,19	*	100,59
Stimulates	Stereotypes_MS	i	1	2,18	0,91	2,4	*	65,2
	Stereotypes VS	HI	−2,57	−1,44	−0,49	2,95	**	209,32
	Attentive object AGO	i	4,15	0,72	0,17	4,21	**	9,79
	Solitary play_SP	HI	−1,09	−2,32	−0,91	2,56	*	244,87
	Instrumentalization/instrumentalized	n	−0,52	2,01	0,97	2,07	*	104,56
**Demand_I**								
	Vocal demand VOCD	I	2,34	0,81	0,33	2,48	*	19,01
	Active response/following proposal_AF	I	5,09	1,78	0,33	5,39	**	19,28
	Proxemic behavior_DPB	I	1	2,18	0,91	2,4	*	65,22
	Joint attention_WA	n	−0,74	3,33	0,98	3,41	**	102,61
	Imitation_NVI	i	0,81	2,35	0,95	2,48	*	70,96
FEXT	Stereotypes_MS	i	2,02	1,87	0,68	2,76	**	42,81
	Instrumentalization/instrumentalized	i	2,28	0,06	0,03	2,28	*	1.47
	Demand_I							
	Vocal demand_VOCD	IV	4,76	−0,4	−0,08	4,77	**	355,15
	Active response/following proposal_AF	i	1,64	2,53	0,84	3,02	**	57,03
	Proxemic behavior_APRO	i	2,15	1,26	0,51	2,5	*	30,41
	Proxemic behavior_DPB	i	1,96	2,85	0,82	3,46	**	55,44
	Joint attention_WO	IV	2,18	−0,4	−0.18	2,22	*	349,53
	Imitation_NVI	IV	2,19	−0,4	−0,18	2,22	*	349,55
GA	Stereotypes_VS	I	4,49	5,02	0,75	6,73	**	48,19
	Autosensori alidad_AS	I	1,72	3,14	0,88	3,58	**	61,25
	Functional intent_FI	I	2,13	1,23	0,5	2,46	*	30,06
	Attentive object AGO	III	−1,97	−0,8	−0,38	2,12	*	202,05
	Blank stare_BS	I	1,68	1,74	0,72	2,42	*	45,91
	Solitary play_SP	I	5,69	4,81	0,65	7,45	**	40,21
	No active response/no following proposal_NAF	III	−1,09	−1,65	−0,84	1,98	*	236,72
	Joint attention DRA	III	−2,12	−1,04	−0,44	2,36	*	206,2
	Joint attention_WA	I	2,64	0,6	0,22	2,71	**	12,79
	Facial expression_FEXR	I	1,77	1,81	0,71	2,53	*	45,53
**Session 16**								
Verbalizes	Stereotypes_MS	n	−0,62	2,21	0,96	2,3	*	105,57
	Stereotypes_VIS	II	−1,06	2,29	0,91	2,52	*	114,88
	Functional intent_FI	HI	−2,73	−0,93	−0,32	2,89	**	198,78
	Blank stare_BS	i	4,52	4,01	0,66	6,04	**	41,55
	Solitary play_SP	HI	−0,72	−1,91	−0,94	2,04	*	249,34
	Normal actions_NA	i	2,82	1,9	0,56	3,4	**	33,99
	Proxemic behavior_APRO	i	1,27	2,58	0,9	2,88	**	63,77
	Joint attention_WA	IV	2.47	−0,5	−0,2	2,52	*	348,56
	Gaze_EC	1	2,09	0,83	0,37	2,25	*	21,6
Vocalizes	Functional intent FI	HI	−2,06	−1,84	−0,67	2,76	**	221,76
	Attentive object_AGO	II	−0,25	2,12	0,99	2,13	*	96,73
	Blank stare_BS	i	2,41	0,65	0,26	2,5	*	15,13
	Normal actions_NA	IV	2,77	−0,09	−0,03	2,77	**	358,09
	Active response/following proposal_AF	I	2,71	0,28	0,1	2,72	**	5,94
	No active response/no following proposal_N7	\l I	1,71	1,71	0,71	2,42	*	45,02
	Gaze_EC	I	0,12	2,28	1	2,28	*	87,04
Verbal imitation	Functional intent_FI	I	2,66	1,7	0,54	3,15	**	32,54
	Solitary play SP	I	2,31	0,37	0,16	2,34	*	9,18
	Instrumentalization/instrumentalized demand_I	I	2,36	2,36	0,71	3,34	**	45,09
	Joint attention_WA	n	−0,94	2,45	0,93	2,62	**	111,06
	Joint attention_WO	II	−0,38	2,36	0,99	2,39	*	99,11
Nonverbal imitation	Solitary play_SP	i	0.14	2,76	1	2,77	**	87,17
	No active response/no following proposal_NAF	IV	2,7	−0,34	−0,12	2,72	**	352,87
Stimulates	Stereotypes_MS	HI	−1,35	−1,46	−0,74	1.99	*	227,31
	Stereotypes_VIS	i	0.62	2	0,95	2,09	*	72,66
	Autosensori alidad_AS	1	0,2	2,28	1	2,29	*	84,9
	Solitary Play_SP	i	1,82	1,3	0,58	2,23	*	35,53
	Active response/following proposal_AF	IV	3,21	−1,23	−0,36	3,44	**	339,05
	Proxemic behavior_APRO	III	−1,67	−1,67	−0,71	2,36	*	225
FEXT	Active response/following proposaLAF	I	0,6	1,99	0,96	2,08	*	73,12
	Joint attention_WO	n	−0,34	2,72	0,99	2,74	**	97,08
GA	Stereotypes_MS	i	2,08	0,85	0,38	2,25	*	22,13
	Stereotypes_VS	i	2,54	2,59	0,71	3,63	**	45,54
	Autosensorialidad_AS	i	2,56	3,07	0,77	3,99	**	50,17
	Functional intent FI	i	2,55	2,54	0,71	3,6	**	44,94
	Attentive object_AGO	i	2.16	2,18	0,71	3,07	**	45,33
	Blank stare_BS	HI	−1,93	−2.46	−0,79	3,13	**	231,82
	Solitary play_SP	i	2,42	4	0,86	4,68	**	58,79
	Normal actions_NA	i	1,31	2,22	0,86	2,58	*	59,52
	Proxemic behavior APRO	1	1,29	1,51	0,76	1,99	*	49,5
**Session 20**								
Verbalizes	Stereotypes_VS	in	−1,73	−1,28	−0,59	2,15	*	216,45
	Erratic behavior_EB	HI	−1,4	−1,38	−0,7	1,97	*	224,51
	Solitary play_SP	i	1,06	1,68	0,85	1,98	*	57,69
	Normal actions NA	HI	−1,82	−1,61	−0,66	2,43	*	221,48
	Vocal demand_VOCD	i	0,31	2.1	0,99	2,12	*	81,52
	Active response/following proposal_AF	i	2,08	1,65	0,62	2,65	**	38,43
	Physical contact_BPC	i	0,46	3,61	0,99	3,64	**	82,76
	Joint attention_DRA	HI	−1,4	−1,38	−0,7	1,97	*	224,51
Vocalizes	Stereotypes_MS	i	3,74	1,52	0,38	4,04	**	22,16
	Solitary play SP	i	2,04	1,16	0,49	2,34	*	29,63
	Joint attention_WA	IV	2,92	−1,41	−0,43	3,24	**	334,26
	Gaze_EC	i	2,26	1,14	0,45	2,53	*	26,79
Verbal imitation	Autosensori alidad_AS	IV	2,69	−0,53	−0,19	2,74	**	348,92
	Blank stare_BS	I	1,65	3,75	0,92	4,1	**	66,32
	No active response/no following proposal_NAF	I	4,08	1,62	0,37	4,39	**	21.64
	Proxemic behavior_APRO	I	1,42	1.41	0,71	2	*	44.89
	Physical contact_BPC	I	6,52	1,15	0,17	6,62	**	10
	Joint attention_DRA	IV	2,31	−0,7	−0,29	2,41	*	343,1
Joint attention_WO		I	1,77	2,84	0,85	3,34	**	58,11
	Gaze_EC	I	0,04	2,53	1	2,53	*	89,15
	Imitation_NVI	I	1,62	7,79	0,98	7,95	**	78,27
Nonverbal imitation	Stereotypes MS	1	3,02	3,39	0,75	4,54	**	48,27
	Stereotypes_VIS	1	1,35	4.14	0,95	4,35	**	71,89
	Normal actions_NA	1	0,61	2.16	0,96	2,25	*	74.12
	Proxemic behavior_APRO	n	−0,6	1,97	0,96	2,06	*	107,07
	Proxemic behavior_DPB	i	2,74	1,12	0,38	2,96	**	22,21
Stimulates	Stereotypes VIS	HI	−1.91	−1,94	−0,71	2,73	**	225,46
	Solitary play_SP	i	1,17	3,53	0,95	3,72	**	71,73
	Instrumentalization/instrumentalized demand_I	i	4,34	0,7	0,16	4,39	**	9,22
	Active response/following proposal_AF	IV	2,4	−0,46	−0,19	2,45	*	349,08
	No active response/no following proposal_NAF	IV	1,69	−1,5	−0,66	2,26	*	318,54
	Proxemic behavior DPB	n	−1,05	2,33	0,91	2,56	*	114,22
	Joint attention_WA	i	3	0,7	0,23	3,08	**	13,2
	Gaze_EC	IV	2,05	−0,89	−0,4	2,23	*	336,37
	Facial expression_FEXR	n	−1,2	2,6	0,91	2,86	**	114,78
FEXT	Stereotypes_VS	IV	2,31	−0,35	−0,15	2,34	*	351,49
	Autosensori alidad_AS	n	−0,45	2,37	0,98	2,41	*	100,72
	Functional intent_FI	i	3,15	3,24	0,72	4,52	**	45,83
	Attentive object AGO	n	−1,12	2,36	0,9	2,61	**	115,36
	Solitary play_SP	i	3,27	0,56	0,17	3,32	**	9,79
	Normal actions_NA	i	1,55	1,44	0,68	2,12	*	42,9
	Joint attention_WO	i	1,91	1,15	0,52	2,23	*	31,08
	Gaze_EC	ni	−1.47	−1,5	−0,71	2,1	*	225,48
GA	Autosensorialidad AS	i	2,18	1,81	0,64	2,83	**	39,65
	Solitary play_SP	in	−1,57	−1,51	−0,69	2,18	*	223,9
	Instrumentalization/instrumentalized Demand_I	HI	−2,59	−0,49	−0,18	2,63	**	190,65
	Proxemic behavior APRO	i	2,72	0,23	0,08	2,72	**	4,77
	Proxemic behavior_DPB	i	2,25	1,7	0,6	2,82	**	37,18
	Joint attention_DRA	i	2,53	1.11	0,4	2,76	**	23,65
	Gaze_EC	i	1,68	2,12	0,78	2,71	**	51,68

Only the conditioned behaviors that generate significant (*) and very significant (^**^) vectors have been selected.

Polar coordinate analysis was carried out using the free program HOISAN 2.0 (Hernández-Mendo et al., 2012), and additionally R (Rodríguez-Medina et al., 2022) in order to obtain a graphic optimization in the vector representation.

In this case study, the focal behaviors were selected from the therapist’s highest frequency categories or dimensions (see Table 1). The analysis was carried out with seven focal behaviors: “Verbalization,” “Vocalization,” “Stimulation” (combining all the categories of each dimension); “Verbal imitation,” “Non-verbal imitation,” “Facial expression” and “Gaze.” In terms of conditioned behaviors, the child’s 28 categories were included from the observation instrument, excluding the codes SAP (sentence approximation) and VD (verbal demand) due to low frequency.

For the analysis, the behaviors of the therapist and the child were recorded in the first 20 min of sessions 9, 16, and 20.

#### Descriptive study of clinically favorable behaviors

From the results obtained in the polar coordinate analysis, the conditioned behaviors (the child’s actions) were selected, that were significantly activated or inhibited prospectively. They were grouped by dimensions of reciprocal social interaction (RSI) or non-reciprocal social interaction (N_RSI).

The dimension “Reciprocal social interaction” consists of the categories: Instrumentalized Demand (I), Non-verbal demand (NVD), Vocal demand (VOCD), Active response (AF), No active response (NAF), Moves closer (APRO), Moves away (DPB), Brief Physical Contact (BPC), Maintained Physical Contact (MPC), Child Shows (SH), Child Draws (DRA), Word Approximation (WA), Word (WO), Gaze (EC), Vocal-verbal Imitation (VI), Non-verbal imitation (NVI), Facial Expression Rejection (FEXR), Facial Expression Joy (FEXJ). The dimension “Non-reciprocal social interaction” consists of the categories: Motor Stereotypes (MS), Vocal Stereotypes (VS), Visual Stereotypes (VIS), Erratic Behavior (EB), Auto Sensory (SA), Functional Intent (INT), Gaze (AGO), Blank Stare (BS), Solitary Play (SP), Normal Actions (NA). The following categories were excluded: NA, referring to normal actions in relation with the therapeutic framework without a sensory purpose, and INT, referring to actions with objects appropriate to the purpose for which they were created.

For each of the therapist’s focal behaviors the percentage of RSI and N_RSI behaviors that were activated and inhibited was calculated. The fact that one of the therapist’s focal behaviors activated RSI behaviors and inhibited N_RSI behaviors was considered clinically favorable. The chi-squared test was used to determine whether the clinically favorable behaviors activated by the therapist were statistically significant, (*p* < 0.05).

## Results and discussion

In the sections below, we describe the relationships detected between interventions by the therapist and the child’s behaviors using polar coordinate analysis and a descriptive study of clinically favorable behaviors.

### Analysis of polar coordinate

Significant results were obtained in activation/inhibition relationships between all the therapist’s and the child’s behaviors.

Table 3 show the level of significance of the focal behavior “Verbalizes,” “Vocalizes,” “Verbal imitation,” “Non-verbal imitation,” “Stimulates,” “FEXT,” “GA” as the main analysis of sessions 9, 16, and 20.

**FIGURE 1:**
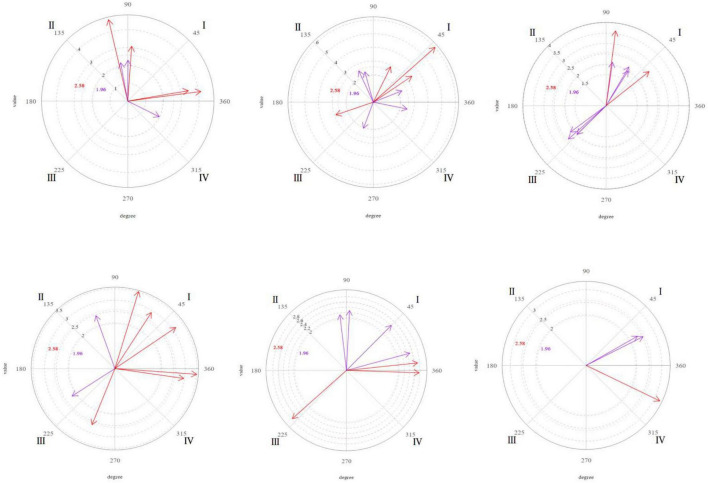
Graphic representation of the significant (purple) and very significant (red) vectors obtained in the polar coordinate analysis. Focal behavior “Verbalizes” as the center of the analysis and all the others as conditional of sessions 9, 16, and 20 (from left to right). Focal behavior “Vocalizes” as the center of the analysis and all the others as conditional of sessions 9, 16, and 20 (from left to right).

**FIGURE 2:**
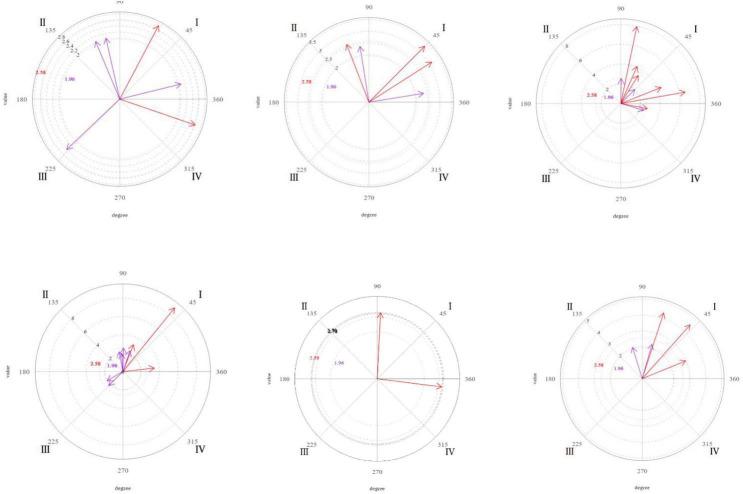
Graphic representation of the significant (purple) and very significant (red) vectors obtained in the polar coordinate analysis. Focal behavior “Verbal imitation” as the center of the analysis and all the others as conditional of sessions 9, 16, and 20 (from left to right). Focal behavior “Non-verbal imitation” as the center of the analysis and all the others as conditional of sessions 9, 16, and 20 (from left to right).

**FIGURE 3:**
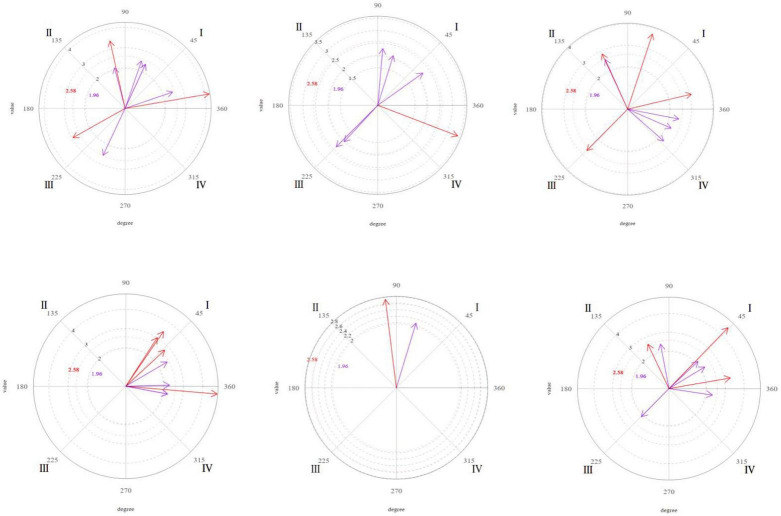
Graphic representation of the significant (purple) and very significant (red) vectors obtained in the polar coordinate analysis. Focal behavior “Stimulates” as the center of the analysis and all the others as conditional of sessions 9, 16, and 20 (from left to right). Focal behavior “Facial expression” as the center of the analysis and all the others as conditional of sessions 9, 16, and 20 (from left to right).

**FIGURE 4:**
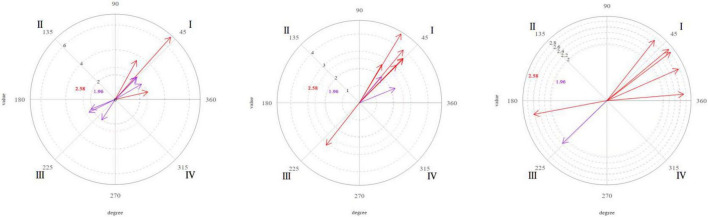
Graphic representation of the significant (purple) and very significant (red) vectors obtained in the polar coordinate analysis. Focal behavior “Gaze” as the center of the analysis and all the others as conditional of sessions 9, 16, and 20 (from left to right).

**Figures 1**–4 shows the significant vectors for all the focal behaviors in each of the therapist’s seven actions.

### Descriptive study of clinically favorable behaviors

Table 4 shows the type and percentage of behaviors that prospectively activated or inhibited each of the therapist’s dimensions.

**TABLE 4:** Type and percentage of behaviors that prospectively activated or inhibited each of the therapist’s dimensions.

Behavior criterion (therapist)	Conditioned behavior (child)			
	
	Activates (I, IV)		Inhibits (II, III)	
		
	RSI	N_RSI	RSI	N_RSI
**Verbalizes**				
s.9	NFI, WO	AGO	NVD	EB
s.16	APRO, EC, WA	BS		MS, VIS, SP
s.20	VOCD, FI, NFI, BPC	SP		VS, EB
*n* (%)	9 (47.3%)	3 (15.8%)	1 (5.3%)	6 (31.6%)
Vocalizes				
s.9	I, NVD, FI, DPB	EB		AGO, VS, SP
s.16	FI, NFI, EC	BS		AGO
s.20	EC, WA	MS, SP		
*n* (%)	9 (52.9%)	4 (23.5%)	0 (0%)	4 (23.5%)
Verbal imitation	RSI	N_RSI	RSI	N_RSI
s.9	NVD, WA	VS	FEXR	AS, SP
s.16	I	SP	WA, WO	
s.20	NFI, APRO, BPC	BS, AS		
*n* (%)	6 (40%)	4 (26.7%)	3 (20.0%)	2 (13.3%)
Non-verbal imitation	RSI	N_RSI	RSI	N_RSI
s.9	NFI, DRA, EC	VS, AS	WO	EB, AGO, SP
s.16	NFI	SP		
s.20	DPB	MS, VIS	APRO	
*n* (%)	5 (33.3%)	5 (33.3%)	2 (13.3%)	3 (20.0%)
Stimulates	RSI	N_RSI	RSI	N_RSI
s.9	VOCD, FI, DPB, INV	MS, AGO	WA, I	VS, SP
s.16	FI	VIS, AS, SP	APRO	MS
s.20	I, FI, NFI	SP	DPB	VIS
*n* (%)	8 (36.6%)	6 (27.3%)	4 (18.2%)	4 (18.2%)
Facial Exp T	RSI	N_RSI	RSI	N_RSI
s.9	I, FI, APRO, DPB, VOCD, WO, INV	MS		
s.16	FI		WO	
s.20		SP, VS		AS, AGO
	8 (57.1%)	3 (21.4%)	1 (7.1%)	2 (14.3%)
Gaze T	RSI	N_RSI	RSI	N_RSI
s.9	WA, EXFI	VS, AS, BS, SP	NFI, DRA	AGO
s.16	APRO	MS, VS, AS, AGO, SP		BS
s.20	APRO, DPB	AS	I	SP
*n* (%)	5 (23.8%)	10 (47.6%)	3 (14.3%)	3 (14.3%)

RSI, reciprocal social interaction; N_RSI, non-reciprocal social interaction; n, number of behaviors.

In the analysis of the child’s clinically favorable behaviors, the therapist’s verbalization was related with 78.9% of clinically favorable behaviors (χ^2^ = 6.53; df = 1; *p* = 0.01) and vocalization with 76.5% (χ^2^ = 5.87; df = 1; *p* = 0.02). Of the therapist’s imitation behaviors, verbal imitation was related with 53.3% (χ^2^ = 0; df = 1; *p* = 1) and non-verbal also with 53.3% (χ^2^ = 0.13; df = 1; *p* = 0.71). Similarly, stimulation with 54.5% (χ^2^ = 0.10; df = 1; *p* = 0.74) and facial expression with 71.4% (χ^2^ = 1.59; df = 1; *p* = 0.20), while gaze produced 38.1% of clinically unfavorable behaviors (χ^2^ = 0.5; df = 1; *p* = 0.47).

In this single case study, we have applied an approach that allows observational methodology and mixed methods as the main analysis (Anguera et al., 2017).

Systematic and meta-analysis reviews show that although child psychoanalytic psychotherapy evidence is increasing, high quality research is needed in order to better understand the effectiveness of such interventions (Midgley and Kennedy, 2011; Abbass et al., 2013; Midgley et al., 2017; Target, 2018). However, there are currently very few centers that systematically evaluate the results of psychoanalytic interventions (Arias-Pujol, 2020). Researchers and therapists, aware of the complexity of each person and the singularity of each psychopathological expression, continue to search for what works and for whom (Fonagy et al., 2016). Recent studies suggest a need to carry out process research in order to identify predictive interaction structures in child psychotherapy (Halfon et al., 2020). At present, different methods and methodologies can be applied, with mixed method methodology representing a new and potentially useful approach for evaluating low level psychoanalytic interventions (Arias-Pujol and Anguera, 2020b).

In the present study, by using a mixed method, we aimed to identify which of the therapist’s techniques elucidated clinically favorable behaviors in a child with severe autism, specifically those that promoted RSI actions and inhibited non-RSI actions. From all of them, verbalization and vocalization by the therapist produced significant clinically favorable behaviors, whereas direct gaze promoted the child’s withdrawal.

Specifically, “Verbalization” (formed by VDE, VOH, VAN, VREM, VSUP, INT) consists of describing a behavior or an object, offering help, anticipating actions happening in the near future, remembering aloud, encouraging or repeating what the child says in a similar way to promote certain dialogue. It is used to bring the child closer to the process of symbolization, following Coromines’ aforementioned psycho-pedagogic technique (Coromines, 1991; Viloca, 1998, 2003; Farrés et al., 2020).

Results show that verbalization activates different behaviors of the RSI dimension related to producing a vocal demand (VOCD), the response to a demand (AF, NAF), proxemic behavior (APRO), eye contact (EC), physical contact (BPC); and in joint attention and also protoconversation behaviors (WA, WO). On the other hand, it inhibits stereotypes (MS, VS, EM, EV, MSI), erratic behavior (EB) and solitary play.

On balance, verbalization is shown to produce significant clinically favorable behaviors in the child, suggesting that this technique is appropriate for activating resources of the child’s RSI dimension. It is of key relevance that verbalization prospectively activates the categories “word” (WO) and “word approximation” (WA), as the child in the study is non-verbal. Results suggest that verbalization promotes language and communication, which are also important in developing the symbolization process.

“Vocalization” is the technique used when the therapist seeks interaction by using exclamations, singing, laughing or encouraging the child to express himself/herself vocally. It has been suggested as especially useful for children with verbal communication difficulties. Results show that use of the “Vocalization” (consisting of EE, SI, L, PF) activates behaviors of vocal and non-vocal demand (I, VOCD); and response to the demand (AF), proxemic behaviors (APRO, DPB), eye contact (EC) and word approximation (WA). It also inhibits the attentive gaze at the object (AGO), the verbal stereotype (VS) and solitary play (SP). Therefore, according to our results, “Vocalization” is an appropriate technique for activating interaction.

Imitation involves mirroring the child’s actions. It has been described as a technique that allows the child to see “outside” in a specific way what should be mentally represented “inside” (Farrés et al., 2020). In this type of intervention, verbal and non-verbal imitation is not used as a way of modeling the child’s behavior but as a way of making contact with the child. Within the framework of mirror neurons theory, the results of a previous study by our group suggest that the systematic use of significant imitation in psychoanalytic psychotherapy with autistic children improves their RSI capacity (Arias-Pujol et al., 2015b).

The present results show that verbal imitation, consisting of VIT, activates the actions of instrumentalization (I), non-verbal demand (NVD), word approximation (WA) and lack of response to the demand (NAF), approximation (APRO) and brief physical contact (BPC). It also inhibits sensory action (SA) and solitary play (SP).

Non-verbal imitation, consisting of NVIT, activates actions of no response to the demand (NAF), drawing (DRA), eye contact (EC) and distancing behavior (DPB). There were some moments when the child appeared to enjoy a kind of dance with music with the therapist. For example, while the child was moving drawing or repeating sounds (ti-ti-ti-ti) he/she gazed with curiosity at how the therapist repeated it. Moreover, non-verbal imitation prospectively inhibited erratic behavior (EB), an attentive gaze at the object (AGO) and the action of solitary play (SP).

However, the behaviors were not statistically significant in the analysis of clinically favorable responses in the child. We believe that these results differ from those of the previous study because on that occasion the use of verbal and non-verbal imitation by the therapist was applied systematically and not depending on the child’s spontaneous behavior.

Stimulation (consisting of SDA, SSH, SDB, SPRO) is used when the child is disconnected from the relationship and the therapist tries to seek his/her attention by giving or showing an object, directing the child’s attention toward something or toward him/herself, or proposing an activity. Results show that “stimulation” activates instrumentalized and vocal demand (I, VOCD), response to the demand (AF, NAF), distancing proxemic behavior (DPB) and the child’s non-verbal imitation (NVI). It also inhibits the attentive gaze at the object (AGO), the blank stare (BS) and solitary play (SP). However, stimulation did not produce statistically significant clinically favorable behaviors. These results suggest that stimulating the child when he/she is very disconnected from the relationship can be more intrusive than verbalizations or vocalizations, and does not always promote reciprocal social interaction.

Dramatization of an emotion consisting of FEXT means making faces or moving the arms and torso, expressing oneself through the body (Viloca, 2003; Farrés, 2014). Results show that it activates behaviors of the RSI dimension. Specifically, in sessions 9 and 16 it activates the instrumentalized and vocal demand (I, VOCD), response to the demand (AF), approximation and distance proxemic behavior (APRO, DPB), the use of words (WO) and the child’s non-verbal imitation (NVI); and prospectively inhibits, in session 20, sensory action (SA) and the attentive gaze at the object (AGO). However, the results did not achieve significance as clinically favorable behaviors.

Finally, direct gaze (consisting of GA) refers to when the therapist is observing the child, allowing him/her to express himself/herself freely. Despite the child’s tendency to disconnect and avoid closeness and physical contact with the therapist, the GA activates the child’s behavior of word approximation (WA), facial expression (FAEX) and of moving close to or away from the therapist (APRO, DPB). Likewise, it inhibits the attentive gaze at the object (AGO), the blank stare (BS) and solitary play (SP). Overall, direct gaze promotes significant withdrawal behaviors, which are not clinically favorable. This result is in line with previous studies suggesting the extreme sensitivity shown by children with autism to small breaks in the therapeutic partnership (Goodman et al., 2017).

## Conclusion

This study shows how observational methodology, and specifically the use of mixed methods, can be useful for the evaluation of a low intensity intervention program. The greatest advantage is that the mixed method perspective allows us to capture the reality just as it happens, systemize it, guarantee its quality and treat it quantitatively in a rigorous way.

From a clinical perspective, our results provide objective evidence – backed up by the application of polar-coordinate-based data analysis – that within a framework of psychoanalytic psychotherapy of a child with severe ASD and no language, the therapeutic techniques of “verbalization” and “vocalization” significantly activate reciprocal social interaction behaviors and inhibit non-social reciprocal behaviors. On the other hand, direct gaze promotes child withdrawal. The results are of key importance as they show the therapist behaviors most useful for promoting social interaction in a child with severe autism.

## Data availability statement

The raw data supporting the conclusions of this article will be made available by the authors, without undue reservation.

## Ethics statement

Written informed consent was obtained from the individual(s), and minor(s)’ legal guardian/next of kin, for the publication of any potentially identifiable images or data included in this article.

## Author contributions

EA-P and MTA contributed to conception and design of the study. MTA conducted the method section and polar coordinate analysis. JM performed the descriptive analysis. EA-P, MM, and JM performed the interpretation of the data. NB adapted and validated the observational instrument. All authors contributed to manuscript revision, read, and approved the submitted version.
